# MvfR Controls Tolerance to Polymyxin B by Regulating *rfaD* in Pseudomonas aeruginosa

**DOI:** 10.1128/spectrum.00426-23

**Published:** 2023-04-11

**Authors:** Fan Yang, Yuchen Zhou, Yuxi Bai, Xiaolei Pan, Un-Hwan Ha, Zhihui Cheng, Weihui Wu, Yongxin Jin, Fang Bai

**Affiliations:** a State Key Laboratory of Medicinal Chemical Biology, Key Laboratory of Molecular Microbiology and Technology of the Ministry of Education, College of Life Sciences, Nankai University, Tianjin, China; b Department of Biotechnology and Bioinformatics, Korea University, Sejong, Republic of Korea; South China Sea Institute of Oceanology

**Keywords:** *mvfR*, polymyxin resistance, LPS, *Pseudomonas aeruginosa*

## Abstract

Polymyxins are currently the last-resort antibiotics for the treatment of multidrug-resistant Gram-negative bacterial infections. To expand the understanding of the intrinsic resistance mechanism against polymyxins, a laboratory strain of Pseudomonas aeruginosa PAO1 was subjected to serial passage in the presence of sublethal doses of polymyxin B over a period of 30 days. By whole-genome sequencing of successively isolated polymyxin B-resistant isolates, we identified a frameshift mutation (L183fs) in the *mvfR* gene that further increased polymyxin resistance in the *pmrB* mutant background. A Δ*mvfR* mutation alone showed higher tolerance to polymyxin B due to altered lipopolysaccharide (LPS) on the surface of bacterial cells, which decreases its outer membrane permeability. In the Δ*mvfR* mutant, polymyxin B treatment caused the upregulation of *rfaD*, the gene involved in LPS core oligosaccharide synthesis, which is responsible for polymyxin tolerance. To the best of our knowledge, this is the first report of *mvfR* mutation conferring polymyxin resistance in P. aeruginosa via increased integrity of bacterial outer membrane.

**IMPORTANCE** Antibiotic resistance imposes a considerable challenge for the treatment of P. aeruginosa infections. Polymyxins are the last-resort antibiotics for the treatment of multidrug-resistant P. aeruginosa infections. Understanding the development and mechanisms of bacterial resistance to polymyxins may provide clues for the development of new or improved therapeutic strategies effective against P. aeruginosa. In this study, using an *in vitro* evolution assay in combination with whole-genome sequencing, we demonstrated that MvfR controls tolerance to polymyxin B by regulating the *rfaD* gene in P. aeruginosa. Our results reveal a novel mechanism employed by P. aeruginosa in the defense against polymyxin antibiotics.

## INTRODUCTION

Polymyxins, such as polymyxin B (PMB) and polymyxin E (colistin), are a group of cationic antimicrobial cyclic peptides (1), some of which are regarded as the last line of defense for the treatment of infections caused by multidrug-resistant (MDR) Gram-negative pathogens, including Escherichia coli, Klebsiella pneumoniae, Pseudomonas aeruginosa, and Acinetobacter baumannii (2–5). Given this fact, there is a pressing need to understand how bacterial pathogens adapt to polymyxin treatment. The development of bacterial resistance to polymyxins is genetically attributed either to mutations in the two-component regulatory systems (TCRs) (PhoPQ and PmrAB) controlling the lipopolysaccharide (LPS)-lipid A pathway (6–8) or to the functional expression of lipid A-modifying enzymes encoded on chromosomes (9) or plasmids (10, 11). Specific mutations in these TCRs can trigger constitutive expression of the *arnBCADTEF*-*pmrE* operon, which encodes enzymes responsible for the covalent attachment of 4-amino-4-deoxy-l-arabinose (l-Ara4N) to lipid A (12–16).

The outer membrane (OM) of Gram-negative bacteria serves as a semipermeable barrier, allowing essential molecules, such as nutrients, to enter the cell while excluding toxic compounds (17). LPS is located on the outer leaflet of the OM and is the major constituent of the Gram-negative cell surface. It is composed of the hydrophobic lipid A, which anchors the LPS to the outer membrane, a core oligosaccharide, and in many species, a repeating distal polysaccharide (O antigen) (18). The bactericidal activity of polymyxins is mediated by an initial charge-based interaction with the lipid A component of LPS. The lipid A produced by most species carries a negative charge due to the presence of free phosphate groups; the binding of positively charged, divalent cations such as Ca^2+^ and Mg^2+^ to the negatively charged phosphate groups stabilizes the LPS (19). However, polymyxins bind these negatively charged phosphate groups with higher affinity than divalent cations and consequently displace Ca^2+^ and Mg^2+^, thus destabilizing the LPS and resulting in reduced OM integrity, leakage of the cytoplasmic content, and eventually, cell death (12, 19). While recent studies have demonstrated that colistin resistance in E. coli and P. aeruginosa is due to modified LPS and protection of the cytoplasmic membrane (CM) rather than the OM from colistin-mediated damage (13, 14), increased LPS transport from the CM to the OM potentiates the effect of polymyxins (15, 20).

Multiple virulence factor regulator (MvfR), also called PqsR, is a LysR-type transcriptional regulator in P. aeruginosa (16) that induces the production of different toxins, such as lectins, pyocyanin, and hydrogen cyanide, and plays a critical role in the virulence of this pathogen (21–23). It directly activates the operons *pqsABCDE* and *phnAB*, resulting in enhanced production of three known intercellular quorum sensing (QS)-related signals, 2-heptyl-4-hydroxyquinoline N-oxide (HQNO), 4-hydroxy-2-heptylquinoline (HHQ), and Pseudomonas quinolone signal (3,4-dihydroxy-2-heptylquinoline [PQS]). MvfR also directly regulates the expression of 34 additional loci across the P. aeruginosa genome, including major regulators and virulence factors, such as the QS regulators *lasR* and *rhlR*, and genes involved in protein secretion, translation, iron homeostasis, and response to oxidative stress (23). In this study, we demonstrate that a mutation in *mvfR* promotes bacterial survival following polymyxin B treatment, as the mutation upregulates *rfaD* expression and alters LPS, leading to reduced OM permeability. Our results reveal a novel mechanism employed by P. aeruginosa in its defense against polymyxin antibiotics.

## RESULTS

### *mvfR* mutation promotes polymyxin B tolerance in P. aeruginosa.

**FIG 1:**
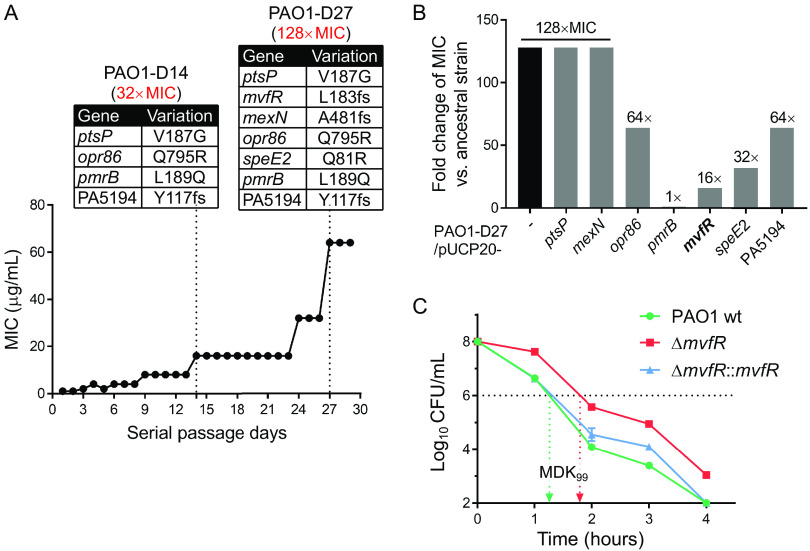
*mvfR* mutation promotes polymyxin B (PMB) tolerance in P. aeruginosa. (A) Resistance acquisition during serial passaging in the presence of sub-MIC levels of PMB in LB medium. All mutations are listed below the corresponding PMB-resistant isolate (PAO1-D14 or PAO1-D27). (B) PMB susceptibility of PAO1-D27 carrying the indicated complementary plasmids. (C) Time-kill curves of PMB against the indicated strains in LB medium. The MDK_99_ is the minimum duration of PMB treatment required to kill 99% of a bacterial population.

To investigate the evolution of polymyxin-resistant mechanisms, P. aeruginosa strain PAO1 was subjected to daily serial passages in the presence of sublethal concentrations of polymyxin B (PMB). The resulting MICs of the culture were recorded daily. After 1 month of evolution, the culture showed a 128-fold increase in the MIC to PMB (Fig. 1A). Isolates with a PMB-resistant phenotype (≥8 μg/mL) (24) were streaked onto drug-free LB plates, and the MICs of individual colonies were then measured. The MICs of PMB-resistant strains PAO1-D14 and PAO1-D27 (isolated on days 14 and 27) were increased 32- and 128-fold, respectively, compared to that of the ancestral strain (Fig. 1A; see Table S1 in the supplemental material). Using the Illumina HiSeq platform, we sequenced the whole genomes of isolates PAO1-D14 and PAO1-D27, as well as those of the control isolates, which were evolved concurrently in PMB-free LB medium and isolated simultaneously. Comparative genomic analysis of the PMB-resistant isolates and the control isolates showed that there were four gene mutations in strain PAO1-D14, a V-to-G change at position 187 encoded by *ptsP* (*ptsP*_V187G_), *opr86*_Q795R_, *pmrB*_L189Q_, and PA5194_Y117fs_ (Fig. 1A; Table S2); three additional mutations were identified in strain PAO1-D27, *mvfR*_L183fs_, *mexN*_A481fs_, and *speE2*_Q81R_ (Fig. 1A; Table S2).

To test the contribution of these substitutions to PMB resistance, the expression vector pUCP20 carrying each wild-type (wt) gene was introduced into the PMB-resistant strain PAO1-D27. Strains harboring the empty vector pUCP20 or complemented with wt *ptsP* and *mexN* genes did not show altered susceptibility to PMB, with a 128-fold MIC increase compared to the wt strain PAO1 (Fig. 1B and Table S1). However, complementation of PAO1-D27 with *opr86* and PA5194 genes reduced the PMB resistance by 2-fold, with *speE2* by 4-fold, and with *mvfR* by 8-fold, while complementation with *pmrB* entirely restored the susceptibility to that of PAO1 (Fig. 1B; Table S1). It is well known that *pmrB* mutation is the primary cause of chromosomally encoded resistance (referred to as endogenous resistance) to polymyxins in P. aeruginosa (12, 25). To the best of our knowledge, the correlation between *mvfR* and susceptibility of P. aeruginosa to polymyxins has not been reported.

To further confirm if the L183fs mutation in *mvfR* confers PMB resistance in P. aeruginosa, we first determined the MIC of an *mvfR* deletion mutant (Δ*mvfR*) to PMB. The MICs of Δ*mvfR* were similar to those of wt PAO1, whether in LB or MH2B medium (Table 1). However, the minimum bactericidal concentration (MBC) of Δ*mvfR* was 2-fold higher than that of wt PAO1 (Table 1). In addition, from the exponential killing curves (Fig. 1C), the minimum duration for killing 99% of bacterial cells in the population (MDK_99_) (26) of strain Δ*mvfR* was obviously longer than the MDK_99_ of wt PAO1. The complemented strain Δ*mvfR*::*mvfR* showed a similar MDK_99_ to that of the wt strain (Fig. 1C), suggesting that *mvfR* mutation promotes the tolerance of P. aeruginosa to PMB.

**TABLE 1:** Susceptibility of different P. aeruginosa strains

Strain	Data for:					
	LB medium		MH2B medium			
	MIC (μg/mL [relative fold change])^*a*^		MIC (μg/mL [relative fold change])		MBC (μg/mL [relative fold change])	
	PMB	Colistin	PMB	Colistin	PMB	Colistin
PAO1 wt (ancestral strain)	0.5	0.5	0.5	0.5	1	0.5
Δ*mvfR*	0.5 (1)	0.5 (1)	0.5 (1)	0.5 (1)	2 (2)	1 (2)
*pmrB*_L189Q_	2 (4)	2 (4)	2 (4)	4 (8)	16 (16)	128 (256)
*pmrB*_L189Q_ Δ*mvfR*	8 (16)	8 (16)	32 (64)	64 (128)	64 (64)	256 (512)
*pmrB*_L189Q_ Δ*mvfR*/pUCP20-*mvfR*	2 (4)	2 (4)	2 (4)	4 (8)	16 (16)	128 (256)
*pmrB*_L189Q_ Δ*mvfR*-Δ*rfaD*	2 (4)	2 (4)	8 (16)	16 (32)	32 (32)	256 (512)
*pmrB*_G188D_	4 (8)	4 (8)	16 (32)	32 (64)	64 (64)	256 (512)
*pmrB*_G188D_ Δ*mvfR*	32 (64)	32 (64)	64 (128)	128 (256)	128 (128)	512 (1,024)
*pmrB*_G188D_ Δ*mvfR*/pUCP20-*mvfR*	4 (8)	4 (8)	16 (32)	32 (64)	64 (64)	256 (512)
*pmrB*_G188D_ Δ*mvfR* Δ*rfaD*	4 (8)	4 (8)	16 (32)	32 (64)	64 (64)	256 (512)

a The fold changes in MIC or MBC relative to wt PAO1 are indicated in parentheses.

### *mvfR* mutation further increases polymyxin resistance in *pmrB* variant backgrounds.

Looking at the evolutionary path to the acquisition of PMB resistance, the mutations *pmrB*_L189Q_ and *mvfR*_L183fs_ occurred successively, and the MIC of PMB increased further after the occurrence of *mvfR*_L183fs_ (Fig. 1A). To confirm if *mvfR* mutation would further increase polymyxin resistance in the *pmrB* mutant background, we introduced *pmrB*_L189Q_ or *pmrB*_G188D_ mutant genes into the Δ*mvfR* background (Table S3); the latter gene is frequently correlated with clinical P. aeruginosa polymyxin resistance (25, 27). The MICs of these strains to both PMB and colistin were determined. The *pmrB*_L189Q_ mutant showed a 4- to 8-fold increase in the MICs of PMB and colistin compared to those of the wt strain (Table 1). The *pmrB*_G188D_ mutation conferred an 8- to 64-fold increase in the MICs of polymyxins compared to those of the wt strain (Table 1). Interestingly, when *mvfR* was further deleted from the *pmrB*_L189Q_- or *pmrB*_G188D_-expressing mutants, the polymyxin MICs were further increased 4- and 16-fold, and complementation with the wt *mvfR* reversed the MICs back to those of single *pmrB* mutants (Table 1). The *pmrB*_L189Q_ or Δ*mvfR* mutations alone did not affect the growth rate of PAO1 cells significantly, while the *pmrB*_L189Q_ Δ*mvfR* double mutant cells grew slightly more slowly than the wt strain cells (Fig. S1). These findings indicate that mutation in *mvfR* further increases polymyxin resistance in P. aeruginosa variants harboring *pmrB* mutations.

### PMB treatment decreases outer membrane permeability in *mvfR* mutants.

**FIG 2:**
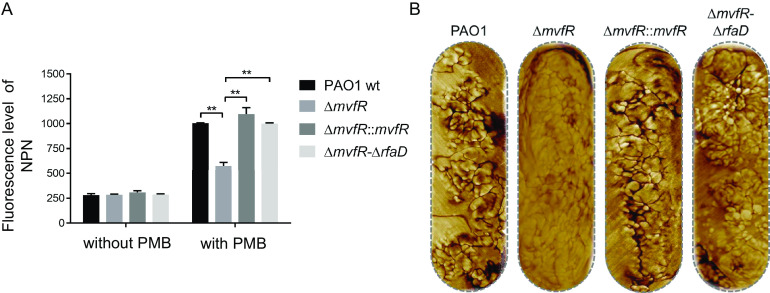
Increased OM integrity of *mvfR* mutant cells in response to PMB treatment. (A) OM permeability in cells of the indicated P. aeruginosa strains grown in LB medium was determined by fluorescent NPN staining. (B) Surface of bacterial cells imaged by atomic force microscopy (AFM). Whole-cell phase images of the indicated strains with 0.75 μg/mL PMB treatment.

As cationic antimicrobial peptides, polymyxins bind to negatively charged lipid A phosphate groups. This destabilizes the OM LPS layer and consequently changes the permeability of the bacterial cell membrane (11, 12). Accordingly, we measured the OM permeability of wt PAO1, the Δ*mvfR* mutant, and a complemented strain (Δ*mvfR*::*mvfR*) using the hydrophobic fluorescent probe 1-*N*-phenylnaphthylamine (NPN) (28). Three strains showed similar OM permeability, with similar levels of NPN uptake without PMB treatment (Fig. 2A). Upon treatment with 0.75 μg/mL of PMB, the OM permeability of all three strains increased; however, compared to wt PAO1 or the complemented (Δ*mvfR*::*mvfR*) strain, the OM permeability of the Δ*mvfR* mutant was significantly decreased (Fig. 2A). These results indicate that the OM permeability of the Δ*mvfR* mutant decreased under the pressure of PMB treatment.

We then asked if the decreased OM permeability under the pressure of PMB in the Δ*mvfR* strain was due to the change in its OM integrity and bacterial cell surface morphology. To address this question, we imaged the entire surface of live bacterial cells using atomic force microscopy (AFM). At a low magnification, the P. aeruginosa cells had a smooth appearance (Fig. S2A, left). The AFM images labeled “phase” represent the variation in the phase of the oscillating AFM probe, which depends on local material properties (29). By recording multiple higher-magnification scans and overlaying them, a map of the accessible OM was obtained, which shows phase-separated LPS patches on the bacterial cell surface (Fig. S2A, right) (30). Consistent with their physiological behavior, the morphology of the Δ*mvfR* mutant cells did not differ significantly from that of wt cells without PMB treatment (Fig. S2B). Nevertheless, the Δ*mvfR* mutant cells showed substantial changes in their OM architecture when they were pretreated with PMB for 30 min; the OM surface looked more intact and smoother, with cracks between the LPS patches significantly reduced and even absent (Fig. 2B and Fig. S2B). Complementation with the wt *mvfR* gene completely reversed the OM morphology to that of wt PAO1 cells (Fig. 2B and Fig. S2B). From microscopic observation, PMB treatment did not cause a size change in the bacterial cells. These results indicate that PMB treatment altered LPS in the OM of Δ*mvfR* mutant cells, thus decreasing their OM permeability.

### Δ*mvfR* causes higher expression of *rfaD* involved in the biosynthesis of LPS core oligosaccharide.

**FIG 3:**
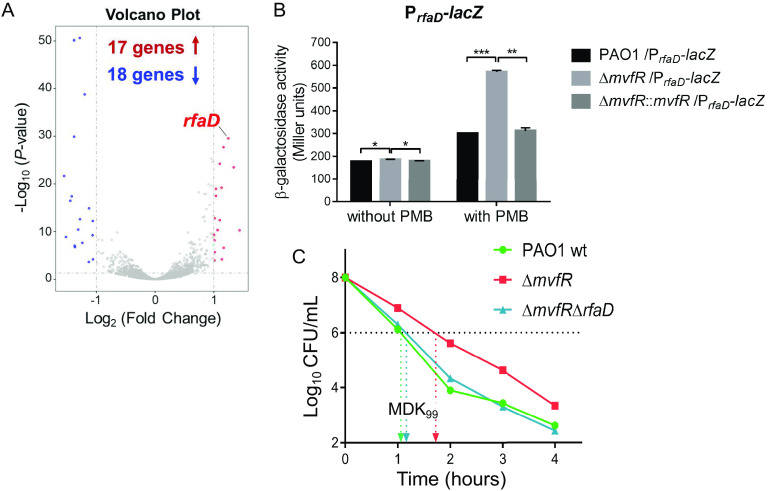
Δ*mvfR* causes higher expression of *rfaD* involved in PMB tolerance. (A) Differential expression of genes responding to PMB treatment in LB medium in Δ*mvfR* versus wt PAO1 cells. Three biological replications of each strain were conducted in RNA-seq experiments. (B) Promoter-*lacZ* fusion assay. P. aeruginosa PAO1 wt, Δ*mvfR* mutant, and complementary Δ*mvfR*:*:mvfR* strains containing the P*_rfaD_*-*lacZ* reporter fusion in plasmid pDN19*lacZΩ* were grown in LB to the late exponential growth phase and analyzed for β-galactosidase activity. Error bars represent the standard deviation (SD). *, *P* < 0.05; **, *P* < 0.01; ***, *P* < 0.001. (C) 1 × 10^8^ CFU/mL of the indicated P. aeruginosa strains was treated with 1 μg/mL PMB in LB medium. At various time points, the cultures were subjected to serial dilution and plating for CFU counting. The MDK_99_ is the minimum duration of treatment that kills 99% of the bacterial population.

To gain insights into the mechanism of LPS layer alteration in *mvfR* mutant cells following PMB treatment, a transcriptomic study was performed on wt PAO1 and Δ*mvfR* mutant cells after PMB treatment for 30 min. Three biological replications of each strain were performed in the transcriptome sequencing (RNA-seq) experiments. The results from these experiments revealed 35 differentially expressed genes between the two strains (≥2.0-fold change in gene expression), excluding the well-known MvfR-regulated target regulons of *phnAB* and *pqsABCDE* (Table S4) (23). A volcano plot in Fig. 3A shows 17 genes upregulated and 18 genes downregulated in the Δ*mvfR* mutant. Among the upregulated genes, *rfaD*, encoding an ADP-l-*glycero*-d-*manno*-heptose 6-epimerase involved in LPS core oligosaccharide biosynthesis (31), was upregulated 2.37-fold compared to the wt strain (*P* = 7.74E−33) (Table S4). A quantitative real-time PCR (q-PCR) was conducted to confirm the expression levels of *rfaD* in the Δ*mvfR* mutant. Consistent with the RNA-seq results, *rfaD* was significantly upregulated in the Δ*mvfR* mutant upon treatment with PMB (Fig. S3A). Moreover, *rfaD* promoter-*lacZ* reporter assays were conducted in the wt PAO1, Δ*mvfR* mutant, and complemented (Δ*mvfR*::*mvfR*) strains. As shown in Fig. 3B, the activity of the *rfaD* promoter in Δ*mvfR* was significantly higher than that in the wt and complemented strains. Direct interaction between the protein MvfR and the promoter DNA of the *rfaD* gene was not detectable by electrophoretic mobility shift assay (EMSA) (Fig. S4). These data suggest that MvfR represses the transcription of the *rfaD* gene indirectly.

We further examined if *rfaD* is involved in the MvfR-mediated control of LPS alteration and OM permeability in P. aeruginosa cells. When the *rfaD* gene was deleted from the Δ*mvfR* mutant, the OM permeability was restored to the wt level (Fig. 2A). From AFM observations, the LPS layer of the Δ*mvfR* Δ*rfaD* double mutant was no longer intact and smooth; instead, scattered LPS patches had appeared, which correlated with reduced OM integrity (Fig. 2B and Fig. S2B), suggesting that the decrease in OM permeability was mainly mediated by *rfaD* upregulation in the *mvfR* mutant.

Regarding antibiotic susceptibility, the Δ*mvfR* Δ*rfaD* double mutant showed a similar MDK_99_ for PMB as wt PAO1 (Fig. 3B), suggesting that the PMB tolerance of Δ*mvfR* was mainly due to the higher expression of *rfaD*. Moreover, deletion of *rfaD* in the *pmrB*_L189Q_ Δ*mvfR* and *pmrB*_G188D_ Δ*mvfR* strain backgrounds restored the MICs of polymyxins to the levels of single *pmrB* mutants (*pmrB*_L189Q_ or *pmrB*_G188D_), respectively (Table 1). These results suggest that *mvfR* mutation confers further polymyxin resistance in a P. aeruginosa PmrB variant background via upregulation of the LPS core biosynthesis gene *rfaD*.

## DISCUSSION

In this study, successive mutational events were identified in P. aeruginosa strain PAO1 during the development of PMB resistance under laboratory conditions. We found that a dysfunctional mutation in the *mvfR* gene can further increase polymyxin resistance in *pmrB* mutants. Mutation of the *mvfR* gene alone in PAO1 did not change its MIC of polymyxins but increased bacterial survival following PMB treatment. The tolerant character of a strain toward an antibiotic can be estimated by determining the MDK_99_, which is the minimum duration of treatment that kills 99% of the bacterial population. Fig. 1C shows that the MDK_99_ of strain Δ*mvfR* was obviously longer than that of the wt strain PAO1 in the presence of PMB at 1 μg/mL, indicating the slower killing of *mvfR* mutant cells; even at a high concentration of PMB, a longer time was required to kill a large fraction of the population (32, 33). *mvfR* mutation promotes the tolerance of P. aeruginosa to PMB. We further demonstrated that PMB treatment induces higher expression of an LPS core biosynthesis gene, *rfaD*, resulting in altered LPS in the OM of Δ*mvfR* mutant cells and consequently decreasing their OM permeability. Deletion of *rfaD* in the Δ*mvfR* mutant background restored its OM permeability and reduced its tolerance to PMB.

The LPS core oligosaccharide has an outer core composed of hexoses and *N*-acetylglucosamine and an inner core composed of the 3-deoxy-d*-manno*-oct-2-ulosonic acid (Kdo) and ADP-l-*glycero*-d*-manno*-heptose (ADP-ld-heptose) residues (34, 35). RfaD, also named HldD or GmhD, catalyzes the conversion of ADP-dd-heptose to ADP-ld-heptose, which is a crucial component of the LPS inner core and connects the outer part of LPS to Kdo between the Kdo_2_ lipid A and O antigen (31, 36). LPS has an important role in maintaining the structural integrity of the OM and providing a physical barrier against the entry of deleterious compounds (37). Escherichia coli K-12 LPS mutants lacking heptose (resulting from a single-site mutation in *rfaD*) are unable to grow in media containing detergents, bile salts, or hydrophobic antibiotics (37, 38). The increased permeability of hydrophobic agents across the outer membrane of heptoseless or other *rfaD* mutants is directly attributed to the loss of the LPS barrier function. Furthermore, the ability of heptoseless or core-defective LPS mutants to survive in the body sites of infected hosts is also compromised (39). Here, the *mvfR* mutant upregulates the expression of *rfaD*, resulting in enhanced tolerance to polymyxin B. Interestingly, an *rfaD* deletion in both the *pmrB*_L189Q_ Δ*mvfR* and *pmrB*_G188D_ Δ*mvfR* strains did not result in a hypersensitivity to PMB. It is possible that P. aeruginosa is able to produce a complete LPS using d-heptose rather than l-heptose, as had been suggested for Neisseria gonorrhoeae (40).

In P. aeruginosa, the *pmrB*_G188D_ variant is frequently correlated with clinical polymyxin resistance (25, 27). The structure of lipid A from a clinical isolate with the *pmrB*_G188D_ gene mutation was determined by mass spectrometry and showed two types of covalent modification in lipid A, including the acyl-oxy-acyl additions of laurate (C_12_:0) by HtrB1 and HtrB2, as well as a single addition of l-Ara4N by ArnT (25). From our experimental results, *mvfR* mutation further increased polymyxin resistance in P. aeruginosa PmrB variants, hinting at a synergistic effect of two distinct polymyxin-resistant mechanisms: (i) lipid A modification caused by *pmrB* mutations (25) and (ii) altered LPS on the outer membrane by increased RfaD in the Δ*mvfR* mutant.

Although this is the first report on the involvement of MvfR in polymyxin resistance, it is possible that *mvfR* mutants have been missed in previous studies of polymyxin-resistant clinical isolates, since *mvfR* mutation alone does not confer polymyxin resistance but rather higher polymyxin resistance to the *pmrB* mutant. As it is likely that prolonged use of polymyxins in the clinic will eventually select for such double mutants, it will be worthwhile to survey clinical isolates with high-level polymyxin-resistant phenotypes for possible mutations in the *mvfR* gene in addition to *pmrB* mutations. Accordingly, proper strategies can be developed to avoid the development of such high-level resistant strains in clinical settings.

In summary, we demonstrated that MvfR controls tolerance to polymyxin B by regulating RfaD in P. aeruginosa. Increased expression of *rfaD* altered the LPS and decreased the OM permeability under the pressure of PMB in the *mvfR* mutant. However, whether the alteration of LPS occurs in the LPS composition, such as the rough/smooth ratio, or the abundance of LPS in the *mvfR* mutant remains for further investigation.

## MATERIALS AND METHODS

### Bacterial strains, plasmids, and growth conditions.

The bacterial strains and plasmids used in this study are listed in Table S2 in the supplemental material. Bacterial cells were grown at 37°C in Luria-Bertani (LB) broth with shaking at 200 rpm or on LB agar plates, if not otherwise indicated. The following concentrations of antibiotics were used: for P. aeruginosa, gentamicin (Gm) at 30 μg/mL, tetracycline (Tc) at 50 μg/mL, and carbenicillin (Cb) at 150 μg/mL; for Escherichia coli, Tc at 10 μg/mL, Gm at 10 μg/mL, kanamycin (Km) at 50 μg/mL, and ampicillin (Ap) at 100 μg/mL. The MIC and minimum bactericidal concentration (MBC) of antibiotics were determined by the 2-fold serial dilution method using LB broth, as well as cation-adjusted Mueller-Hinton broth (MH2B), as indicated. Susceptibility was interpreted according to the Clinical and Laboratory Standards Institute guidelines (24). All experiments were conducted in the biosafety level 2 laboratory at Nankai University.

### Experimental evolution for selecting PMB-resistant mutants.

We performed sequential passages for a month of P. aeruginosa PAO1 in the presence of sublethal levels of PMB to obtain resistant mutants. Overnight cultures of strain PAO1 were divided into three technical replicates, each grown in LB only or LB containing 0.5× MIC, 1× MIC, 2× MIC, or 4× MIC of PMB. At 24-h intervals, the cultures were checked for growth. Cultures from the highest concentration that allowed growth to an optical density at 600 nm (OD_600_) of ≥2 were diluted 1:100 into fresh media containing higher PMB concentrations. This serial passaging was repeated daily for 30 days. The cultures were passaged on drug-free LB agar plates, and the MIC was determined using the broth microdilution method. The bacterial cells were then cryopreserved with 15% glycerol for revival later.

### Whole-genome sequencing and reference mapping.

Bacterial genomic DNA was extracted with DNA purification kit (Tiangen Biotech, Beijing, China). Fragments smaller than 500 bp were obtained from 200 ng genomic DNA by sonication (Covaris S220), followed by end treatment and adaptor ligation. Adaptor-ligated DNA fragments of about 470 bp were recovered using beads and then PCR amplified for six cycles; the PCR products were cleaned up using beads and quantified using a Qsep100 machine (BiOptic, Taiwan, China) and a Qubit 3.0 fluorometer (Invitrogen). Sequencing was carried out using a 2 × 150-bp paired-end (PE) configuration on an Illumina HiSeq instrument according to the manufacturer’s instructions. The data were aligned to the PAO1 reference genome (GenBank accession number NC_002516.2) via BW2 version 0.7.12 software. Single nucleotide variations (SNVs) or indel mutations were detected using SAMtools version 1.1 software and the Unified Genotyper module from GATK.

### Determination of bacterial tolerance to PMB.

Overnight bacterial cultures were diluted 1:100 in fresh LB medium and grown to an OD_600_ of 1.0. Bacterial cells were collected by centrifugation and resuspended in LB to reach a concentration of 1 × 10^8^ CFU/mL. After being treated with 1 μg/mL PMB at 37°C for 1, 2, 3, and 4 h, the bacterial cells were subjected to serial dilution and plating for CFU enumeration. Three parallel samples of the same strain were subjected to CFU determination. According to the time-kill curves, the MDK_99_ (the minimum duration for killing 99% of a bacterial population) for the tolerant strain should be higher than the MDK_99_ for the susceptible strain (26).

### Assessment of outer membrane permeability.

The integrity of the outer membrane was analyzed by the fluorescent probe 1-*N*-phenylnaphthylamine (NPN; Beijing Ouhe Technology). Briefly, bacterial cells cultured overnight were diluted 1:100 into fresh LB medium and cultured at 37°C to the late log phase (OD_600_ = 1). The cells were washed with a 5-mM HEPES buffer containing 5 mM glucose (GHEPES). The bacterial cell suspension was standardized to an OD_600_ of 0.5 in the GHEPES buffer. NPN was added to the cells at a final concentration of 10 μM. The PMB was added into the bacterial suspension at a concentration of 0.75 μg/mL. The fluorescence was monitored at excitation/emission wavelengths of 350/420 nm with a luminometer (Varioskan Flash; Thermo Scientific). All tests were conducted in triplicate.

### Atomic force microscopy.

Freshly grown bacterial cells were harvested at 12,000 rpm for 2 min and resuspended in minimal medium (1× M9 salt, 2 mM MgSO_4_, and 0.4% glucose). A drop of bacterial suspension was placed onto the cover glass and fixed by flame heating. AFM assay was performed using the Bruker Dimension Icon platform, with an OTESPA probe with a driving frequency of 140 to 300 kHz. The spring constant was 42 N/m. Tapping mode was used for the AFM imaging. The AFM images were recorded at a line frequency of 2 to 8 Hz, 500 nm, and 512 pixels square.

### RNA-seq and data analysis.

Both wt PAO1 and *mvfR* mutant cultures (OD_600_ = 1) were pretreated with 0.75 μg/mL polymyxin B for 30 min in LB medium. Total RNA was isolated using an RNAprep pure cell/bacteria kit (Tiangen Biotec). Three replicates were prepared for each strain. Sequencing and analysis were performed as previously described (41).

### Statistical analysis.

Statistical analyses were performed using GraphPad software. Student’s (two-tailed) *t* test was utilized to assess the statistical significance of two-group comparisons. Data were considered significant at *P* values of less than 0.05, as indicated.

### Data availability.

The transcriptome (RNA-Seq) data have been deposited at NCBI GenBank under the BioProject accession number PRJNA845932.
