# Supplementary material for: MvfR Controls Tolerance to Polymyxin B by Regulating rfaD in Pseudomonas aeruginosa
Source: Microbiol Spectr. 2023 Apr 11;11(3):e00426-23. doi: 10.1128/spectrum.00426-23 (PMC10269820; doi:10.1128/spectrum.00426-23)
Supplement: Supplemental file 1 — Supplemental material. Download spectrum.00426-23-s0001.pdf, PDF file, 0.7 MB [file spectrum.00426-23-s0001.pdf]

Supplemental Information

MvfR Controls Tolerance to Polymyxin B by Regulating *rfaD* in *Pseudomonas aeruginosa*

Fan Yang[1], Yuchen Zhou[1], Yuxi Bai[1], Xiaolei Pan[1], Un-Hwan Ha[2], Zhihui Cheng[1], Weihui Wu[1], Yongxin Jin[1*], Fang Bai[1*]

[1] State Key Laboratory of Medicinal Chemical Biology, Key Laboratory of Molecular Microbiology and Technology of the Ministry of Education, College of Life Sciences, Nankai University, Tianjin 300071, China.
[2] Department of Biotechnology and Bioinformatics, Korea University, Sejong 30019, Republic of Korea.

*, Corresponding authors: Fang Bai (baifang1122@nankai.edu.cn) and Yongxin Jin (yxjin@nankai.edu.cn)

This file contains

1) **Supplementary Table S1-S5.**
2) **Supplementary Figure 1-4.**
3) **Supplementary Methods**
4) **References**

Supplementary Tables

Table S1. Susceptibility of polymyxin B (PMB)-resistant *P. aeruginosa* isolates.

Strains	MIC (μg/mL) [a]	
	in LB medium	in MH2B medium [b]
PAO1 wt (ancestral strain)	0.5 (1)	0.5 (1)
PAO1-D14	16 (32)	32 (64)
PAO1-D27	64 (128)	128 (256)
PAO1-D27/pUCP20	64 (128)	128 (256)
PAO1-D27/pUCP20-*ptsP*	64 (128)	128 (256)
PAO1-D27/pUCP20-*mexN*	64 (128)	128 (256)
PAO1-D27/pUCP20-*opr86*	32 (64)	128 (256)
PAO1-D27/pUCP20-*pmrB*	0.5 (1)	2 (4)
PAO1-D27/pUCP20-*mvfR*	8 (16)	16 (32)
PAO1-D27/pUCP20-*speE2*	16 (32)	128 (256)
PAO1-D27/pUCP20-PA5194	32 (64)	128 (256)

[a] The fold changes in MIC relative to wt PAO1 strain were indicated in parentheses.

[b] MH2B: cation-adjusted Mueller Hinton broth.

Table S2. Comparative genomic analysis between PMB-resistant *P. aeruginosa* isolates and the control isolates [a].

Type [b]	Start	End	Ref [c]	Obs [d]	Gene	AAChange [e]	Products
PMB-resistant isolate **PAO1-D14**							
INDEL	5847479	5847479	T	-	PA5194	Y117fs	hypothetical protein
SNV	379157	379157	T	G	PA0337	V187G	phosphoenolpyruvate-protein phosphotransferase PtsP
SNV	4085072	4085072	T	C	PA3648	Q795R	outer membrane protein Opr86
SNV	5365325	5365325	T	A	PA4777	L189Q	two-component system signal sensor kinase PmrB
PMB-resistant isolate **PAO1-D27**							
INDEL	1086549	1086549	G	-	PA1003	L183fs	LysR-type transcriptional regulator MvfR (PqsR)
INDEL	1565409	1565410	TT	-	PA1436	A481fs	RND efflux transporter MexN
INDEL	5847479	5847479	T	-	PA5194	Y117fs	hypothetical protein
SNV	379157	379157	T	G	PA0337	V187G	phosphoenolpyruvate-protein phosphotransferase PtsP
SNV	4085072	4085072	T	C	PA3648	Q795R	outer membrane protein Opr86
SNV	5362387	5362387	A	G	PA4774	Q81R	spermidine synthase SpeE2
SNV	5365325	5365325	T	A	PA4777	L189Q	two-component system signal sensor kinase PmrB

[a] The control isolates were evolved in PMB-free LB from the same ancestral strain, and isolated simultaneously with the PMB-resistant isolates on day-14 and day-27.

[b] Data from Illumina HiSeq mediated reference genome PAO1 (NC_002516.2) comparison. Synonymous mutations and mutations in the non-coding regions were excluded. SNV, single nucleotide variant; INDEL, insertion-deletion

mutations. [c] Base type of reference genome. [d] Sample genome base type. [e] Amino acid change.

Table S3. Bacterial strains and plasmids used in this study.

Strains/plasmids	Description	Source
P. aeruginosa		
PAO1	Wild-type	Lab stock
PAO1-D14	Serial passage of PAO1 for 14 days under the sub-MIC of polymyxin B	This study
PAO1-D27	Serial passage of PAO1 for 27 days under the sub-MIC of polymyxin B	This study
Δ*mvfR*	*mvfR* (ΔL183) deletion mutant of PAO1	This study
Δ*mvfR*::*mvfR*	*mvfR* self-promoter driven *mvfR* inserted into the chromosome of Δ*mvfR* strain via mini-Tn7	This study
Δ*pmrB*	*pmrB* deletion mutant of PAO1	This study
$pmrB_{L189Q}$	$pmrB_{L189Q}$ variant inserted into the chromosome of Δ*pmrB* strain via mini-Tn7	This study
$pmrB_{G188D}$	$pmrB_{G188D}$ variant inserted into the chromosome of Δ*pmrB* strain via mini-Tn7	This study
$pmrB_{L189Q}$-Δ*mvfR*	*mvfR* deletion mutant of $pmrB_{L189Q}$ strain	This study
$pmrB_{G188D}$-Δ*mvfR*	*mvfR* deletion mutant of $pmrB_{G188D}$ strain	This study
$pmrB_{L189Q}$-Δ*mvfR*-Δ*rfaD*	*mvfR*-*rfaD* double mutant of $pmrB_{L189Q}$ strain	This study
$pmrB_{G188D}$-Δ*mvfR*-Δ*rfaD*	*mvfR*-*rfaD* double mutant of $pmrB_{G188D}$ strain	This study
E. coli		
DH5α	For general cloning and sub-cloning; *lacZ*ΔM15, *recA1*, *endA1*	Lab stock
S17	Donor strain for conjugation	Lab stock
Plasmids		
pUCP20	Shuttle vector between *E. coli* and *P. aeruginosa*; Ap^r, Cb^r	(1)
pUCP20-*pmrB*	*pmrB* gene from PAO1 in pUCP20; Ap^r, Cb^r	This study
pUCP20-*mvfR*	*mvfR* gene from PAO1 in pUCP20; Ap^r, Cb^r	This study
pUCP20-*ptsP*	*ptsP* gene from PAO1 in pUCP20; Ap^r, Cb^r	This study
pUCP20-*mexN*	*mexN* gene from PAO1 in pUCP20; Ap^r, Cb^r	This study
pUCP20-*opr86*	*opr86* gene from PAO1 in pUCP20; Ap^r, Cb^r	This study
pUCP20-*speE2*	*SpeE* gene from PAO1 in pUCP20; Ap^r, Cb^r	This study
pUCP20-PA5194	PA5194 gene from PAO1 in pUCP20; Ap^r, Cb^r	This study
pEX18Tc	Gene replacement vector, $oriT^+$, $sacB^+$; Tc^r	(2)
pEX18Tc-*pmrB*	*pmrB* gene deletion on pEX18Tc; Tc^r	This study
pEX18Tc-*mvfR*	*mvfR* gene L183fs deletion on pEX18Tc; Tc^r	This study
pEX18Tc-*rfaD*	*rfaD* gene deletion on pEX18Tc; Tc^r	This study
P_{rfaD}-*lacZ*	*rfaD* promoter cloned into pDN19-*lac*Ω; Tc^r	This study
pMAL-c2x	MBP-tag protein expression vector, Ap^r	This study
pMAL-c2x-MvfR	The coding region of *mvfR* cloned into pMAL-c2x, Ap^r	This study
pMAL-c2x-$MvfR_{1-183}$	The N-terminal 1-183 amino acids of MvfR cloned into pMAL-c2x, Ap^r	This study

pUC18T-mini-Tn7T-Gm	mini-Tn7 base vector from insertion into chromosome *att*Tn7 site, Gm^r	(3)

Table S4. Differential expression of genes responding to polymyxin B (Δ*mvfR* vs wt PAO1 strain)[a]

Gene Name	Regulation	Fold Change	*P* value	Product
nrdG	Up	2.71	5.32E-13	class III (anaerobic) ribonucleoside-triphosphate reductase activating protein NrdG
PA5207	Up	2.52	1.38E-26	phosphate transporter
rfaD	Up	**2.37**	7.74E-33	ADP-L-glycero-D-mannoheptose-6-epimerase
PA2077	Up	2.24	3.87E-09	hypothetical protein
PA3278	Up	2.24	6.10E-31	hypothetical protein
PA1918	Up	2.19	1.80E-06	hypothetical protein
PA3613	Up	2.19	2.89E-22	hypothetical protein
PA4328	Up	2.14	2.28E-27	hypothetical protein
ccoN2	Up	2.14	3.54E-15	cbb3-type cytochrome C oxidase subunit I
PA2118a	Up	2.09	4.65E-13	hypothetical protein
PA5232	Up	2.05	5.24E-22	hypothetical protein
PA4571	Up	2.05	1.87E-20	cytochrome C
ccoQ2	Up	2.02	3.83E-08	cytochrome C oxidase cbb3-type subunit CcoQ
ccoP2	Up	2.02	1.30E-15	cytochrome C oxidase cbb3-type subunit CcoP
PA3282	Up	2.02	3.53E-06	hypothetical protein
PA5208	Up	2.02	7.56E-11	hypothetical protein
PA1789	Up	2.01	5.37E-12	hypothetical protein
PA3519	Down	2.08	1.82E-06	hypothetical protein
PA4387	Down	2.09	5.19E-15	phage exclusion suppressor FxsA
PA3808	Down	2.09	6.10E-12	hypothetical protein
fdx2	Down	2.18	8.83E-18	(2Fe-2S) ferredoxin
PA5023	Down	2.19	8.07E-06	hypothetical protein
hscB	Down	2.30	4.04E-42	co-chaperone HscB
PA5024	Down	2.36	2.95E-10	hypothetical protein
PA3522	Down	2.43	2.14E-15	resistance-nodulation-cell division (RND) efflux transporter
hscA	Down	2.44	5.01E-54	chaperone protein HscA
PA1847	Down	2.48	3.62E-13	Fe/S biogenesis protein NfuA
PA1856	Down	2.58	2.60E-09	cbb3-type cytochrome C oxidase subunit I
PA3523	Down	2.59	1.35E-09	resistance-nodulation-cell division (RND) efflux membrane fusion protein
iscR	Down	2.60	3.21E-33	HTH-type transcriptional regulator
iscA	Down	2.60	1.62E-53	iron-binding protein IscA
PA3574a	Down	2.68	2.43E-20	copper chaperone CopZ
iscU	Down	2.72	2.15E-19	scaffold protein
PA3518	Down	2.87	1.45E-11	hypothetical protein

iscS	Down	2.93	8.49E-25	cysteine desulfurase
phnB	Down	8.73	1.19E-113	anthranilate synthase component II
phnA	Down	18.04	1.36E-250	anthranilate synthase component I
pqsE	Down	24.47	9.99E-177	thioesterase PqsE
pqsD	Down	45.42	1.25E-252	3-oxoacyl-ACP synthase
pqsC	Down	109.78	0	hypothetical protein
pqsB	Down	121.92	0	hypothetical protein
pqsA	Down	145.12	0	anthranilate--CoA ligase

[a] Three biological replications of each strain were conducted in RNA-seq experiments. The last seven genes (*phnB* to *pqsA*) are known direct regulons of MvfR.

Table S5. Primers used in this study.

**Primer**	**Sequence (5' to 3')**	**Purpose**
rfaD-F	CGGCGACACCGACATCA	qRT-PCR
rfaD-R	TCCAGCAGGCGGCAACT	
ppiD-F	CGCTGAAGCAAGGTGAGGTA	qRT-PCR
ppiD-R	GCTTTCCAGGCTCGGTACTT	
pmrB-F	TATGACCATGATTACGAATTCATGTCCCGTGCCGCCGTC	*pmrB* complementation
pmrB-R	ACGACGGCCAGTGCCAAGCTTTCAGATATGTGACCGCCCGCTG	
opr86-F	TATGACCATGATTACGAATTCATGAAACGCTTTCTGCTACCCGC	*opr86* complementation
opr86-R	ACGACGGCCAGTGCCAAGCTTTCAGAAGGTCTGGCCCAGGGAG	
PA5194-F	TATGACCATGATTACGAATTCATGGATAATGCCTCTCCGTTCCAAG	PA5194 complementation
PA5194-R	ACGACGGCCAGTGCCAAGCTTTCAGGCCGAGTCGCGGAC	
mvfR-F	TATGACCATGATTACGAATTCATGCCTATTCATAACCTGAATCACGTG	*mvfR* complementation
mvfR-R	ACGACGGCCAGTGCCAAGCTTCTACTCTGGTGCGGCGC	
speE2-F	TATGACCATGATTACGAATTCGTGCCGGCGGAGGTCG	*speE2* complementation
speE2-R	ACGACGGCCAGTGCCAAGCTTTCAGGTCCTCGCCAGGGCC	
ptsP-F	TATGACCATGATTACGAATTCATGCTCAACACGCTGCGC	*ptsP* complementation
ptsP-R	ACGACGGCCAGTGCCAAGCTTTCAGGGCTGGACGGTAGCC	
mexN-F	TATGACCATGATTACGAATTCGTGACGCCGCGCGCC	*mexN* complementation
mexN-R	ACGACGGCCAGTGCCAAGCTTTCATGTCGCCGTCG	

	CCCC	
*mvfR*_up-F	TATGACCATGATTACGAATTCGATTCTAACCGCATAGGTCGCGC	*mvfR* ΔL183 deletion
*mvfR*_up-R	GGCCAGCGTCTGCTGGAGAACGGAGGCATTGCACAACGGGTG	
*mvfR*_down-F	CACCCGTTGTGCAATGCCTCCGTTCTCCAGCAGACGCTGGCC	
*mvfR*_down-R	ACGACGGCCAGTGCCAAGCTTAGGTGCGCGACATGCTCAAG	
*rfaD*_up-F	TATGACCATGATTACGAATTCCACGTCCACCGGGATGTTGTC	*rfaD* deletion
*rfaD*_up-R	CTGACCCTGCTGCCGGAGCTGCGATGAATGCCCCCAGGCTATGC	
*rfaD*_down-F	GCATAGCCTGGGGGCATTCATCGCAGCTCCGGCAGCAGGGTCAG	
*rfaD*_down-R	ACGACGGCCAGTGCCAAGCTTGGCGTGTCCTTCGCCGCGATCG	
*pmrB*_up-F	TATGACCATGATTACGAATTCGGCAACAACCATGCGTTCAGC	*pmrB* deletion
*pmrB*_up-R	CTTCGCGTAGCGGCGCCTGGCCGCGGGCAACGCCGAACCAG	
*pmrB*_down-F	CTGGTTCGGCGTTGCCCGCGGCCAGGCGCCGCTACGCGAAG	
*pmrB*_down-R	ACGACGGCCAGTGCCAAGCTTGAACACCTGCACTTCCAGC	
pmrB$_{189}$_up-F	TATGACCATGATTACGAATTCGGCAACAACCATGCGTTCAGC	*pmrB* $_{L189Q}$
pmrB$_{189}$_up-R	CTTCGCGTAGCGGCGCCTGGCCGCGGGCAACGCCGAACCAG	
pmrB$_{189}$_down-F	CTGGTTCGGCGTTGCCCGCGGCCAGGCGCCGCTACGCGAAG	
pmrB$_{189}$_down-R	ACGACGGCCAGTGCCAAGCTTGAACACCTGCACTTCCAGC	
pmrB$_{188}$_up-F	TAATTCGATCATGCATGAGCTCCTGCGCCTGGAAGGCGATACC	*pmrB* $_{G188D}$
pmrB$_{188}$_up-R	CACTTCGCGTAGCGGCGCCAGGTCGCGGGCAACGCCGAAC	
pmrB$_{188}$_down-F	GTTCGGCGTTGCCCGCGACCTGGCGCCGCTACGCGAAGTG	
pmrB$_{188}$_down-R	TTCGCGAGGTACCGGGCCCAAGCTTCCTATCCCTTTCCCGGCCAATG	
rfaD.F	TTAAAACGACGGCCAGTGAATTCCGAGGGCGGACAGGGGAAGCAG	*rfaD* promoter-LacZ reporter assay

rfaD-R	TATCTAGAACCTCCTTAGGATCCCTCCCTTCGACAGGATGAATGCCCC	
pMAL-MvfR-F	GAGGGAAGGATTTCAGAATTCATGCCTATTCATAACCTGAATCACGTGAACATGTTC	MBP-MvfR expression
pMAL-MvfR-R	ACGACGGCCAGTGCCAAGCTTCTACTCTGGTGCGGCGCGCTGG	
pMAL-MvfR-F	GAGGGAAGGATTTCAGAATTCATGCCTATTCATAACCTGAATCACGTGAACATGTTC	MBP-MvfR$_{1-183}$ expression
pMAL-MvfR$_{1-183}$-R	ACGACGGCCAGTGCCAAGCTTGTAATTGGCCAGGCTCGCGATGC	
rfaD-F	CGAGGGCGGACAGGGGAAGCAGGAG	EMSA probe
rfaD-R	TCCCTTCGACAGGATGAATGCCC	
pqsA-F	GAAGCCTGCAAATGGCAGGCGAGG	EMSA probe
pqsA-R	GACAGAACGTTCCCTCTTCAG	

Supplementary Figures

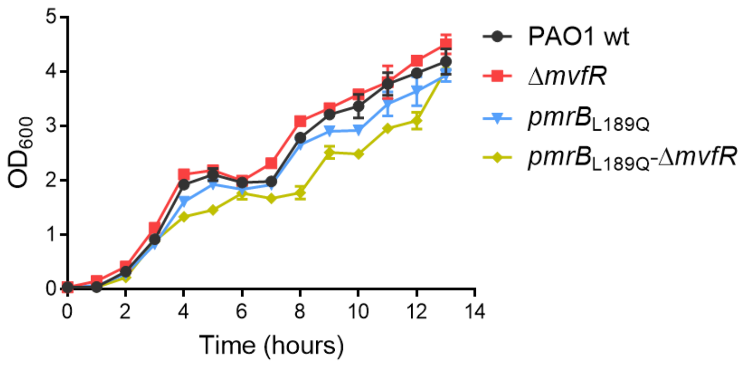

Figure S1. Growth curves of different *P. aeruginosa* strains. Cultures were grown in LB liquid medium at 37 °C with constant agitation (200 rpm) on a rotary shaker and the OD_{600} measured at various time points.

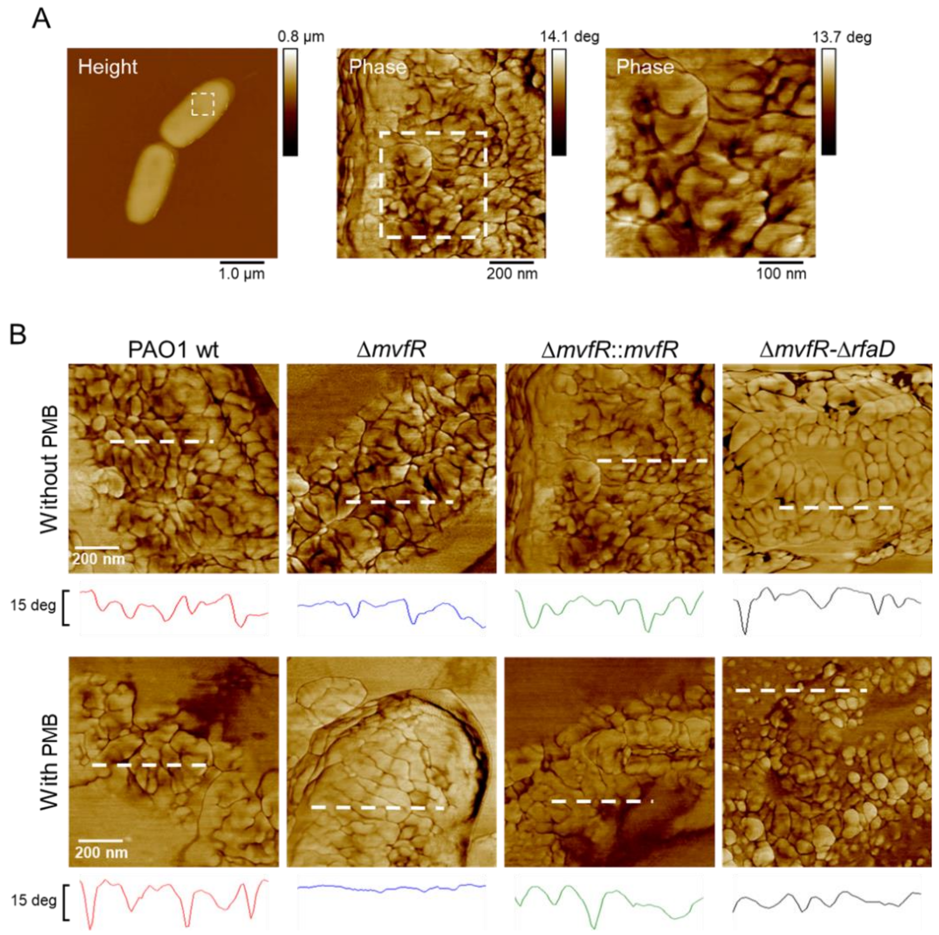

Figure S2. Surface of bacterial cells imaged by atomic force microscopy (AFM). (A) Height and phase images of cell surface of *P. aeruginosa* PAO1 strain at different magnifications. (B) Phase images of OM surface of indicated strains with or without 0.75 μg/mL PMB treatment. Color curves below indicate phase profiles of dashed lines in the above AFM images.

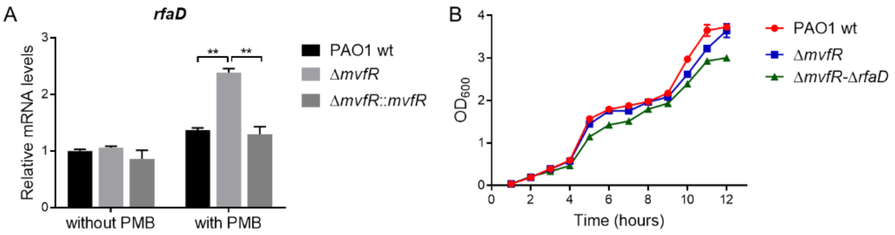

Figure S3. High expression of *rfaD* in Δ*mvfR* mutant leading to increased LPS amount. (A) q-PCR detection of *rfaD* expressing levels in indicated strains with/without 0.75 μg/mL polymyxin B (PMB) treatment. (B) Growth curves of indicated strains in LB liquid medium at 37 °C on a rotary shaker. Error bars represent SD. *, *P*<0.05; **, *P*<0.01.

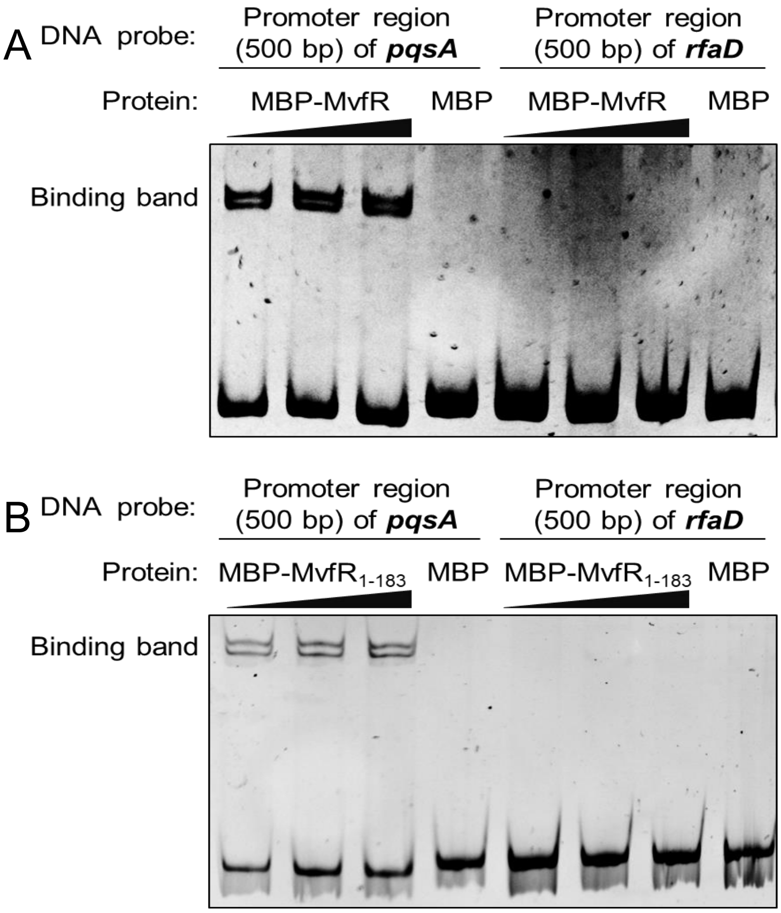

Figure S4. Electrophoretic mobility shift assays (EMSA) Determination of the bingding ability of full-length MvfR protein (A) and N-terminal DNA-bingding domain MvfR$_{1-183}$ (B) to the promoter region of *rfaD* gene. *pqsA* promoter region is the positive control. MBP tag alone is the negative control.

Supplementary Methods

Protein expression and purification. The DNA fragments encoding the full-length and the truncated (1 to 183 aa) MvfR were amplified using the specific primers (Supplementary Table S5) cloned into pMAL-c2x to express recombinant MvfR (MBP-MvfR) and $MvfR_{1-183}$ (MBP- $MvfR_{1-183}$), respectively. Escherichia coli BL21 (DE3) cells were transformed with the constructed plasmids to express MBP-MvfR and MBP-$MvfR_{1-183}$. The E. coli strains were induced to express the recombinant proteins with 1 mM isopropyl-thio-β-D-galactoside (IPTG; Solarbio, Beijing, China) at 22°C for 16 h. The recombinant proteins were purified from soluble fractions using MBP Sep Dextrin Agarose Resin 6FF (Yeasen, Shanghai, China) following the manufacturer's instruction.

Electrophoretic mobility shift assay (EMSA). DNA fragments corresponding to the promoter regions of *rfaD* and *pqsA* were synthesized by GENEWIZ (Suzhou, China). Fifty ng of DNA probe were incubated with 0 to 6 µg purified MBP-MvfR, MBP-$MvfR_{1-183}$ or MBP (negative control) at 16 or 25°C for 30 min in a binding buffer (4 mM Tris-HCl [pH 8.0], 4% glycerol,2 mM dithiothreitol [DTT; Solarbio], 4 mM $MgCl_2$, 40 nM NaCl. and 2.5 ng/µL salmon sperm DNA) in a final volume of 20 µL. Samples were loaded onto an 8% native polyacrylamide gel in 1 × TBE buffer (89 mM Tris-borate [pH 8.3], 2 mM EDTA), which had been prerun for 1 h at 100 V, and electrophoresed at 10 mA on ice for 1.5 h. The gel was stained in 1× TBE containing 0.5 µg/ml ethidium bromide at room temperature for 10 min. Bands were visualized using a ChemiDoc™ XRS+ molecular imager (Bio-Rad).

References

1. Hoffmann L, Sugue MF, Brüser T. 2021. A tunable anthranilate-inducible gene expression system for Pseudomonas species. Appl Microbiol Biotechnol 105:247-258.
2. Hoang TT, Karkhoff-Schweizer RR, Kutchma AJ, Schweizer HP. 1998. A broad-host-range Flp-FRT recombination system for site-specific excision of chromosomally-located DNA sequences: application for isolation of unmarked Pseudomonas aeruginosa mutants. Gene 212:77-86.
3. Choi KH, Schweizer HP. 2006. mini-Tn7 insertion in bacteria with single attTn7 sites: example Pseudomonas aeruginosa. Nat Protoc 1:153-61.
